# Riociguat for pulmonary arterial hypertension (PAH) associated with congenital heart disease (CHD): A subgroup analysis from the PATENT studies

**DOI:** 10.1186/2050-6511-16-S1-A79

**Published:** 2015-09-02

**Authors:** Rosenkranz Stephan, Hossein-Ardeschir Ghofrani, Maurice Beghetti, Dunbar Ivy, Arno Fritsch, Gerrit Weimann, Soundos Saleh, Christian Apitz, Reiner Frey

**Affiliations:** 1 Department III of Internal Medicine and Cologne Cardiovascular Research Center (CCRC), Cologne University Heart Center, Cologne, Germany; 2 Department of Internal Medicine, University Hospital Giessen and Marburg, Giessen, Germany; 3 Paediatric Cardiology Unit, Children's Hospital, University Hospital of Geneva, Geneva, Switzerland; 4 Department of Pediatrics, University of Colorado School of Medicine, Children's Hospital Colorado, Aurora, Colorado, US; 5 Bayer Pharma AG, Wuppertal, Germany; 6 Pediatric Heart Centre, University Children's Hospital, Giessen, Germany

## Background

Pulmonary arterial hypertension associated with congenital heart disease (PAH-CHD) is a common associated form of PAH [1]. Riociguat, a soluble guanylate cyclase stimulator, was shown to be a safe and effective treatment for patients with PAH in the Phase III PATENT-1 study and the PATENT-2 long-term extension [2,3]. Here we report data for the subgroup of patients with PAH-CHD in PATENT-1 and PATENT-2.

## Materials and methods

In PATENT-1, adults with symptomatic PAH were randomized to receive placebo, riociguat up to 2.5 mg three times daily (tid) or riociguat up to 1.5 mg tid (exploratory) for 12 weeks. Patients completing PATENT-1 without ongoing riociguat-related serious adverse events (AEs) were eligible to enter the PATENT-2 long-term extension, during which all patients received open-label riociguat up to 2.5 mg tid.

## Results

**Figure 1.**
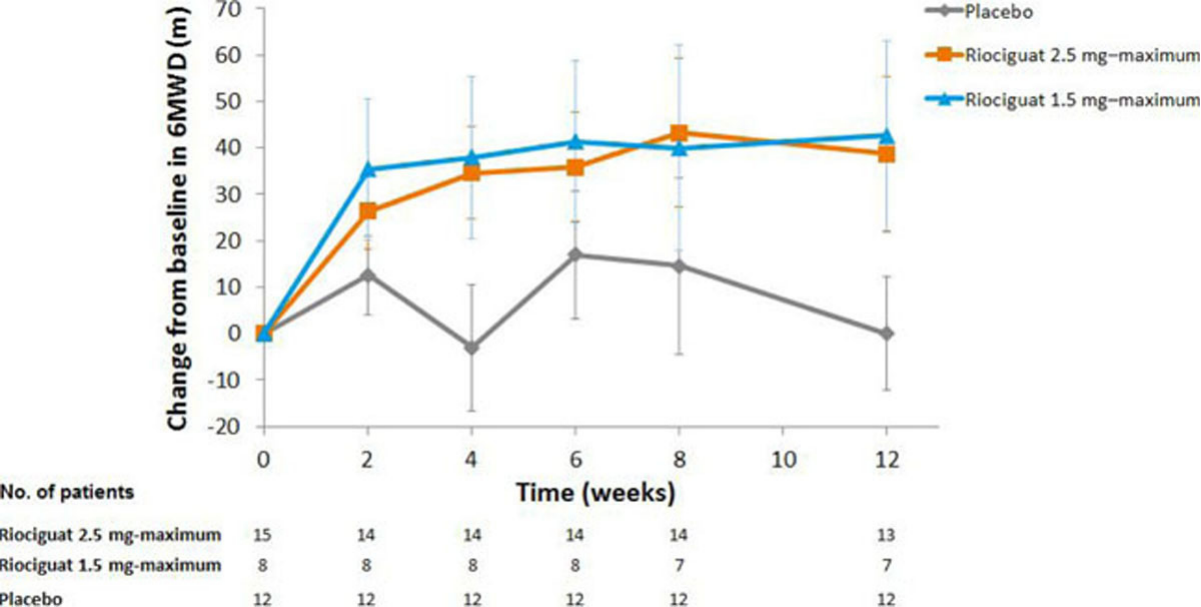
Change from baseline in 6MWD in patients with PAH-CHD in PATENT-1 Data are observed values (mean±SEM) 6MWD, 6-minute walking distance; SEM, standard error of the mean

**Figure 2.**
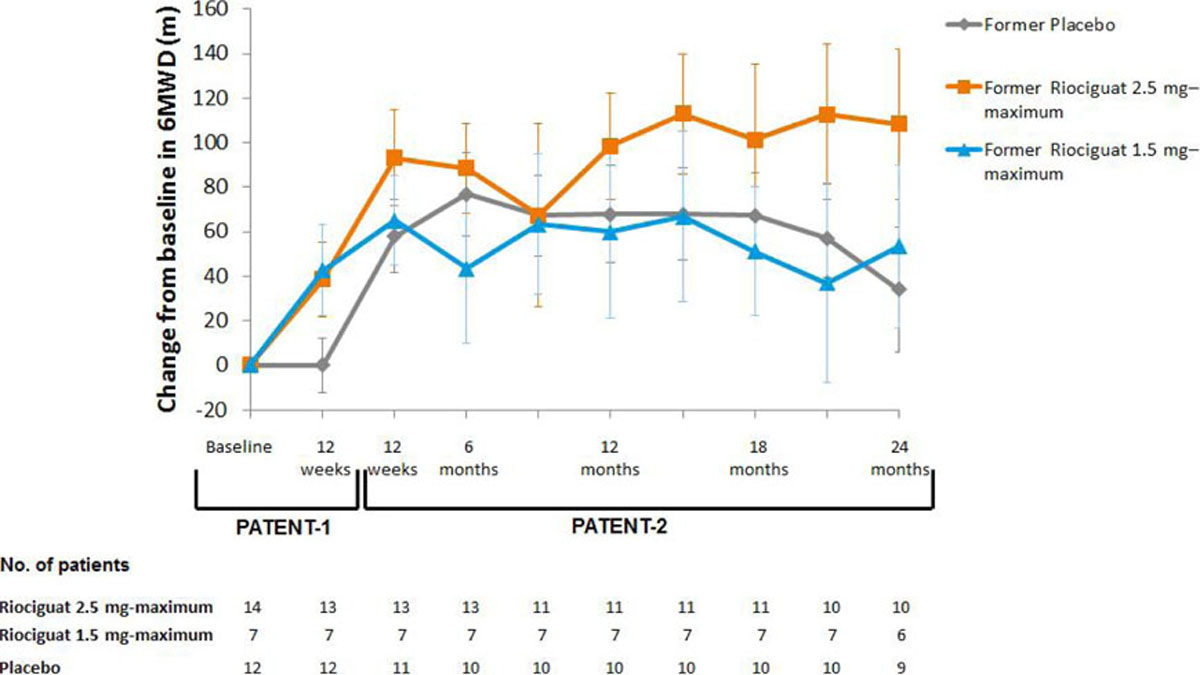
Change from baseline in 6MWD in patients with PAH-CHD in PATENT-1 and PATENT-2 Data are observed values (mean±SEM) Data from PATENT-1 are shown for patients who subsequently entered PATENT-2 6MWD, 6-minute walking distance; SEM, standard error of the mean

There were 35 patients with PAH-CHD in PATENT-1. All had persistent/recurrent PAH following complete surgical repair of CHD. Mean time since last corrective surgery was 16.8 years. At baseline, 57% of patients were treatment-naïve and all were in WHO FC II (60%) or III (40%). At Week 12, 6MWD (primary endpoint) had increased from baseline by mean±SD 39±60 m in the riociguat 2.5 mg–maximum group and by 43±54 m in the 1.5 mg–maximum group and was unchanged with placebo (Figure 1). Several secondary endpoint, including PVR, NT-proBNP and WHO FC, also improved from baseline in both riociguat groups (Table 1). Of 35 patients with PAH-CHD in PATENT-1, 33 entered PATENT-2. The improvements in 6MWD with riociguat seen in PATENT-1 were sustained for up to 2 years; 6MWD also increased in the former placebo group after transition to riociguat (Figure 2). At 2 years the overall mean±SD change from PATENT-1 baseline in 6MWD in PATENT-2 was +68±97 m and WHO FC had improved/stabilized/worsened in 32/60/8% of patients (n=25). In PATENT-1, the most commonly reported AEs (occurring in ≥15% of PAH-CHD patients in any treatment group) were dyspepsia, headache, dizziness, palpitations, back pain, nausea, vomiting, chest discomfort, dyspnea and pain in extremity. Six serious AEs were reported in these patients in PATENT-1: intra-abdominal hemorrhage (one riociguat 2.5 mg–maximum patient); right ventricular failure and worsening PAH (separate events in one 1.5 mg–maximum patient); loss of consciousness (one placebo patient); pneumothorax and supraventricular tachycardia (separate events in one placebo patient). None of these events were considered related to study drug. No new or unexpected safety signals were observed in PAH-CHD patients in PATENT-2.

**Table 1:** Changes from baseline to end of Week 12 in secondary endpoints in patients with PAH-CHD in PATENT-1 (observed values):

	Placebo			Riociguat 2.5 mg–maximum			Riociguat 1.5 mg–maximum		
	
	n	Baseline	Change from baseline	n	Baseline	Change from baseline	n	Baseline	Change from baseline
PVR (dyn·s·cm–5)	11	1312±763	-66±632	13	1130±664	-250±410	7	1047±564	-126±368

NT-proBNP (pg/mL)	12	1573±1775	-46±697	13	761±1172	-164±317a	7	1352±1350	-872±1147a

WHO FC (%)	12	II – 58%	Improved 8%	15	II – 67%	Improved 21%	8	II – 50%	Improved 29%
		III – 42%	Stabilized 83%		III – 33%	Stabilized 79%		III – 50%	Stabilized 71%
			Worsened 8%			Worsened 0%^a^			Worsened 0%^a^

Borg dyspnea score	12	4.3±2.7	-0.1±2.4	15	2.5±1.4	-0.3±1.3^b^	8	3.2±1.6	-0.8±0.8^a^

EQ-5D score	12	0.74±0.16	-0.05±0.22	15	0.78±0.15	0.03±0.18^a^	8	0.74±0.08	+0.09±0.14^a^

LPH score	12	40.4±20.0	-0.1±15.8	15	34.9±26.0	-8.0±15.9^a^	8	40.0±15.3	-13.7±13.2^a^

Data are mean±SD unless otherwise indicated.

^a^ Data missing for one patient.

^b^ Data missing for two patients. EQ-5D, EuroQol Group 5-Dimension Self-report Questionnaire; Living with Pulmonary Hypertension questionnaire NT-proBNP, *N*-terminal prohormone of brain natriuretic peptide; PVR, pulmonary vascular resistance; WHO FC, World Health Organization functional class

## Conclusion

This exploratory subgroup analysis showed that riociguat improved clinical outcomes and was well tolerated in patients with persistent/recurrent PAH following complete surgical repair of CHD.
